# A Qualitative Exploration on the Challenges of Transfer Students in an Asian Educational Context

**DOI:** 10.3390/ijerph18063238

**Published:** 2021-03-21

**Authors:** Shirley Siu Yin Ching, Kin Yuen Tam, Lillian Weiwei Zhang, Jeremy Ng, Kin Cheung

**Affiliations:** 1 School of Nursing, The Hong Kong Polytechnic University, Yuk Choi Road, Hung Hom, Kowloon, Hong Kong, China; shirley.ching@polyu.edu.hk (S.S.Y.C.); lillianzhangweiwei@163.com (L.W.Z.); jeremyntd@gmail.com (J.N.); 2 Department of Applied Social Sciences, The Hong Kong Polytechnic University, Yuk Choi Road, Hung Hom, Kowloon, Hong Kong, China; kin.yuen.tam@polyu.edu.hk

**Keywords:** transfer students, academic adjustment, social integration, self-identity, psychological well-being, higher education in Asia

## Abstract

Limited research has been conducted on community college (CC) transfer students’ (TS) experiences in four-year universities, particularly in Asian contexts. To fill this research gap, in this qualitative study, 124 TS from various disciplines in a Hong Kong university participated in 39 focus groups and seven individual interviews. Unlike their Western counterparts, our TS were relatively better prepared and more academically adaptive. Nevertheless, their social integration was restricted by a lack of time for extra-curricular activities, a sense of inferiority and incompetence, and restricted social circles that did not enable interaction with non-TS. These challenges and their implications are discussed. In particular, this study has highlighted differences between the special education systems for CC transfer in Hong Kong and those in Western CC models. The study has also highlighted the study-induced stress, and poor self-perceptions that TS experience, despite their academic abilities.

## 1. Introduction

Following the massification of higher education, community college (CC) transfer has emerged as a common route of entry to baccalaureate studies, as an alternative to direct post-secondary admission [1,2]. Due to the growing aspirations of CC graduates to pursue bachelor’s degrees in university (also known as “four-year institutions” in the literature), the size of the CC transfer student (TS) population has been booming over the past decade [1,3]. This calls for more attention to CC TS through identifying their characteristics and needs [4,5,6,7,8,9,10,11]. Although there is no shortage of quantitative studies on this subpopulation of university students, particularly in the West, few qualitative studies have been conducted worldwide to understand their transfer experiences.

The highly complex transition processes brought about by transfer can bring about a “turbulent pathway” (p. 600) [11] to TS, challenging their psychological, academic, and environmental adjustment [4,5,7,11,12]. Driving a host of studies on CC TS [13], Astin’s theory of student involvement [14] and Tinto’s model of student integration [15] posit that both academic and social involvement and integration into the university contribute to students’ persistence in learning and their degree attainment. Transfer student capital, as a synthesis of various concepts such as social capital and cultural capital [16], refers to the factors bringing about successful transition to university, including but not limited to the processes by which knowledge and experience are acquired by them during baccalaureate studies [17]. In the existing TS literature, it has remained understudied in how TS capital is manifest and influential on the TS’ learning experience in universities.

A commonly known phenomenon confronting TS is “transfer shock”, defined as the drop in post-transfer GPA compared to previous academic performances in CC [10,18]. Transfer shock is usually attributed to a lack of academic preparation in their prior studies [19] and personal factors such as family and work commitments [20]. TS were, therefore, found to be more susceptible to attrition [21]. They might have heavier study loads than non-TS [22] and need to demonstrate active and independent learning approaches [13]. As for social involvement, TS were found to have insufficient interactions with their peers [22] and to engage less than non-TS in social groups and activities [1]. Exclusive to TS is a phenomenon coined as “campus culture shock” that can occur when faced with the less personal atmosphere and support systems in university as opposed to those in CC [23,24].

Prior studies have suggested that university management and administrators lack a sound understanding of CC TS’ experiences, leading to “institutional neglect” of these students as a separate population and thereby “overlooking” (p.174) [25] their needs and the resources available to them [25,26]. Aside from the pool of aforementioned quantitative studies, Tobolowsky and Cox [26] suggested that a qualitative inquiry into TS’ experiences and perceptions would allow not only a probing of their thoughts and feelings but also some unique examples and anecdotes. In fact, identifying the challenges encountered by TS as an integral part of the university can lead to improved understanding of student mobility and persistence [27]. Flaga [28] interviewed 35 TS from various majors, yielding that they perceived the academic environment of the university as “demanding but fair”, that their integration into the social environment happened in a later timeframe than their non-transfer counterparts, and that they were aware of the more “decentralized” campus services and department functions of the university when compared to CC. In particular, they reported the fast-paced progression of courses demonstrated the university’s high academic standards, and that they were inclined towards seeking informal resources (e.g., peers) as support rather than formal channels (e.g., faculty) [28]. Later, after interviewing 57 students, Owens [27] concluded that the TS had expected the academic atmosphere of the university to be more sophisticated, yet still feeling “intimidated” by the workload. They found it difficult to focus on their studies if they joined extracurricular activities and were concerned they were not “fitting into” the university culture, thus feeling “overwhelmed” and “completely alone”. In addition to academic and social aspects, Owens [27] also suggested an identity-related issue, with TS having a feeling of “marginality” in their university classes. Townsend and Wilson [29] conducted interviews with 19 students and suggested that TS were more interested than non-TS in the academic and social activities that would be helpful for their degree attainment. Another interview-based study with 11 TS also showed their emphasis on academic engagement and indifference towards building social networks [25].

Most of the studies that examined TS’ experiences have been conducted in Western educational contexts, predominately in the U.S., with small samples, and within limited majors. As well, most were conducted more than ten years ago. Furthermore, it is not uncommon for TS in Western countries to be more mature [30], racially or ethnically more diverse [31], and having had some full-time work experience prior to baccalaureate studies [32]. Despite being much less researched, CC transfer also takes place in such Asian countries and regions as mainland China, Hong Kong, and Malaysia [33,34]. Notably, the landscape of higher education in Asia might differ in that there is relatively less emphasis on racial diversity [35], and more on being “elite institutions” [36]. Academic qualifications and post-secondary employability seem to be more emphasized and pursued in Asian societies than in the West [37]. While it is increasingly common to earn post-secondary credentials (e.g., Associate Degrees) in Greater China [38], in Hong Kong these qualifications are mandatory requirements for CC graduates to transfer to university [33]. In particular, while CC TS in the West can normally spend several years of studies for obtaining their baccalaureate degrees, those in Hong Kong are normally required to complete their studies in two years [33]. Furthermore, in Hong Kong CC are largely perceived as the stepping stone for entering university [39,40], as opposed to CC in other parts of the world that primarily aim at democratizing and improving the affordability of higher education [41,42]. Pioneering quantitative research on TS in the Asian educational context, the authors [5,6,7] have conducted an institution-wide questionnaire survey in the university with the largest number of TS in Hong Kong, yielding that TS tend to experience obstacles with credit transfer, to experience transfer shock, to have heavier study loads and mental health issues, to be less active in participating in social and extra-curricular activities, and to show self-stigmatization. However, not much is known about the current learning experiences and challenges of TS across various disciplines. Moreover, compared to prior studies in the West [25,43], there is a lack of discussion on transfer pathways and articulation policies in the Asian education context, which can be reflected from delving into CC TS’ first-hand experience. Thus, we put forward the research question: In an Asian educational context, what are the challenges in CC TS’ experiences in university? The results will contribute to the TS literature, and also inform local and global stakeholders about institutional policies and practices, including senior management, student affairs offices, and academic advisors to tackle the transition issues and thus enhance the psychological well-being of the TS. As Asian students make up a considerable population in higher education all over the world, this study can also inform those receiving institutions of practices in supporting Asian TS as the target student population.

## 2. Methods

This study adopted a qualitative approach using focus group with TS on a designated topic (i.e., learning experiences and challenges in university study) in an informal setting [44]. Focus groups were chosen because they generate conversations that uncover individual opinions and reveal group consensus on their experience. They facilitate the interviewees to discuss potentially complex phenomena in a more amenable setting and reveal what they thought and why they thought what they did [45].

### 2.1. Research Context

Under normal circumstances, high school leavers who fail to meet the university entrance requirements on the public examination can enroll in local CCs that provide an “alternative route to higher education” (p. 147) [46]. Different from CC transfer in Western countries, a CC graduate in Hong Kong not only needs a competitive GPA, but also to obtain an associate degree (AD) or higher diploma (HD) in order to articulate vertically to bachelor’s degree studies [47]. These CC TS are often admitted to university as third-year students, and they are normally required to complete their baccalaureate studies full-time in two years (i.e., third and fourth years of the university).

### 2.2. Participants

Purposive sampling was used to recruit participants from a university in Hong Kong. The inclusion criteria were TS who had (a) graduated from a two-year CC, and (b) been studying in the bachelor’s degree program for at least one semester, or (c) graduated from the program within one or two years. The research assistant sent invitation emails to target participants through the university’s internal email system and invited those who replied to the emails to join the interview. Of the 124 interviewees, 84 (67.7%) were female. Sixty-one (49.2%) were in their first year of study, 45 (36.3%) in their second year, 9 (7.3%) in their third year, and 9 (7.3%) were graduates. Following the categorization of disciplines according to the Qualifications Framework ( http://www.hkqr.gov.hk/HKQRPRD/web/hkqr-en (accessed on 19 March 2021), 45 (36%) were from Medicine, Dentistry and Health Sciences (MDHS); 19 (15%) from Business and Management (BM); 17 (14%) from Services (SV); 11 (9%) from Architecture and Town Planning (ATP); 11 (9%) from Engineering and Technology (ET); 7 (6%) from Languages and Related Studies (LRS); 6 (5%) from Sciences (SS); 5 (4%) from Arts, Design and Performing Arts (ADPA); and 3 (2%) from Computer Science and Information Technology (CSIT).

### 2.3. Data Collection

Ethical approval was obtained (HSEARS20170808003) prior to conducting the interviews. The focus groups were conducted in a quiet room in the university and moderated by an interviewer with substantial experience in qualitative research. In addition to the interviewer, a research assistant joined each interview and noted the interactions among the participants. Students from the same program were interviewed as a group.

All focus groups were conducted in Cantonese, the commonly used language in Hong Kong after obtaining informed consent from each interviewee. An interview guide was developed (Table 1). A funnel-based approach [48] was adopted; the interviews started with the broad question “Can you tell me about your experience studying in the university till now?” followed by probing questions that allowed the interviewees to elaborate on their experiences and challenges. The interviewer facilitated the discussion on the topic, which was essential for themes from the interviewees’ opinions to emerge during data collection and analysis [49]. After each interview, the interviewer and the research assistant discussed the key themes, hunches, interpretation, and what was new to the “data pool” and made minor adjustments to the interview questions whenever necessary.

Owing to availability of the students, seven individual interviews were conducted in the university by the same interviewer using the focus groups guide. The funnel-based approach [48] was adopted. Each student was asked to share their experience in studying in university at the beginning of the interview. The interviewer then asked probing questions through which the students elaborated more on their transition experience, learning methods, and experience with other students. Interviews were completed when the students do not have any further comments to add.

### 2.4. Data Analysis

The audio recordings of the interviews were transcribed verbatim, word-by-word, in Chinese. The data were then imported into NVivo Pro 12 and coded for subsequent analysis using the inductive approach. Manifest qualitative content analysis facilitated us to have a broad and in-depth understanding of the phenomenon in question [50]. According to Graneheim and Lundman [51], qualitative content analysis consists of five steps: (i) read and re-read the interview transcriptions, (ii) identify the meaning units (sentences/ paragraphs) corresponding to different aspects of the students’ experiences, (iii) condense meaning units (i.e., excerpts) and label with codes, (iv) identify subcategories by comparing their similarities and differences, and (v) delineate the key categories. Through an iterative coding process where refinements were made from multiple rounds of going through the data, a coding framework was developed (Table 2). The interviewer and the research assistant served as two independent coders. First, they coded the excerpts from the first 10 interviews (21.7% of 46 interviews) separately. They then discussed obtaining a consensus on the coding framework before adopting it to analyze the subsequent interviews, which involved two more research assistants. Intermediate summarizing and peer debriefings were conducted throughout the data analysis to ensure the consistency of the data analysis. Updates of the coding framework were made to match the evolving understanding of the phenomenon. The trustworthiness of the study was established through an audit trail that contained the raw data, field notes, intermediate summaries, process notes, and coding frameworks [52]. The strategies to ensure the trustworthiness of the findings have been described in other papers [8,9].

## 3. Results

Thirty-nine focus groups and seven individual interviews, depending on their availability, were conducted with 124 TSs from various disciplines and years of study from February 2018 to March 2019. The interviews consisted of one to five interviewees and lasted for 90–150 min. The categories and codes are summarized in Table 2. Overall, the excerpts can be categorized as those related to the challenges with learning, issues of self-identity, and social experience. At the end of each quote presented in this paper, the interview identifier (#1 representing Interview 1), the interviewee identifier (e.g., S1 representing Interviewee 1), the discipline of interviewees (e.g., SV for Services, as shown above), and the interviewee’s year of study (e.g., Year 1 indicating first year) are displayed.

### 3.1. Challenges in Learning Experiences

As competition for admission to university is keen, the TS studied hard in CCs to aim for good academic performances. Some TS continued with the same mode of learning even after transferring to university, thus being motivated in their academic pursuits.

*We were very serious when studying in the associate degree program. Some of us may have kept on with this mode of learning till now [in university].*

(#33, S91, SV, Year 1)

*We pay much more effort in studying than non-TS. We know very well what we want to achieve… We have been used to fighting for a high GPA. We try our best to study and have an in-depth understanding [of knowledge] and mastery of the practical skills.*

(#30, S80, ET, Year 1)

#### 3.1.1. Heavy Workload

Perception of foreshortened period of bachelor study. With some credits transferred from the CC to their degree programs, TS’ studies can be shortened from four to an average of two to three years [33]. Logically, completing the study in a shorter period of time is an advantage of the transfer study pattern. However, because of the perceived foreshortened period of baccalaureate studies, the TS anticipated the need to catch up and had to deal with packed schedules.

*The non-TS are at an advantage as they have more time. They study two more years [than us] in the university.*

(#2, S7, MDHS, Year 2)

*We are different from non-TS. We focus more on academic performance. Studying is our priority. Actually, we are just like chasing an outstanding debt. That is, we have to catch up with the course content. The non-TS are more relaxed [than us] and they can manage both study and other activities.*

(#28, S76, MDHS, Graduate)

The TS generally reported having heavy study loads. They felt unsure if they would achieve better academic performances despite devoting most of their time and effort to this pursuit. They did not feel any sense of control over their own study, but needed to manage their study-induced stress.

*After studying hard for two years in CC, I want to relax a bit when studying at university and graduate with an acceptable academic performance … We seize the time to study hard in university. However, we can no longer finish all the tasks like we did in [CC].*

(#4, S14, MDHS, Year 2)

*We need to manage the stress from studying because the workload is relatively high. Moreover, I expect to achieve a good academic performance. It is necessary to find myself some ways to learn better.*

(#3, S8, SV, Year 2)

Limited by insufficient credits transferred and extra workload from non-major courses. The majority of the students expressed that the graduation requirements for general education courses, along with service learning, contributed to heavy workloads. This was especially the case for students who had few credits transferred. They also pointed out having to take courses similar to those from their CC studies, in order to fulfil the graduation requirements.

*I was only able to transfer the credits of two courses [from the CC to degree programmes]. As a result, I have to take six courses in each semester. I was basically studying all the time in my first year in university. It affected me a lot as I had no time for other activities.*

(#25, S71, MB, Year 2)

*There were similar courses [between the CC and university], yet we failed to have the credits transferred. Then, we had to take those courses again. The contents were almost the same… It’s a waste of time.*

(#3, S9, SV, Year 2)

Due to their heavy workloads, the students suggested having credit transfer for more non-major courses.

*The biggest challenge for me was fulfilling the requirements of the general education courses and service learning.*

(#1, S1, MDHS, Year 2)

*The requirements for language and general education courses may be different in various institutions. Students may be required take these courses again even if they studied similar courses in CC. Their workloads could then be much heavier. I think having to take the courses related to our major is fine. Perhaps we could “waive” more for courses unrelated to the major.*

(#32, S87, ATP, Year 2)

Inflexible arrangement of time because of timetabling. The students commented that they have difficulty managing their time because of timetabling. Most of the courses for the TS were pre-assigned by their departments. In addition, due to limited vacancies in some courses and inflexible class timeslots, students might not always be able to enroll in their desired courses or classes. Subsequently, some of them had to give up taking some courses they were interested in or even gave up participating in overseas exchange programs.

*Some courses are offered to the TS only. Therefore, there are limited choices of timeslots. We may waste the time in between lessons. The timetabling does not allow us to plan according to our needs and we do not take enough rest.*

(#46, S124, SS, Year 2)

*TS face more restrictions in timetabling. We have to complete all requirements for general education courses within two years … Some students need to choose another service-learning course instead of the one they are interested in because of the time clashes. The purpose of facilitating personal development [in a university] was defeated.*

(#30, S83, ATP, Year 1)

Dearth of time for extracurricular activities. The majority of the students claimed that managing the time spent on different aspects was a formidable challenge. They had great expectations for active involvement in university life but reflected the reality that it was difficult to strike a balance between studying and extracurricular activities.

*I want to maintain a balance between studying and engaging in extracurricular activities. I yearn to participate in some activities in the University.*

(#34, S92, ADPA, Year 1)

*I wish to have memories of the university life that are more than just studying.*

(#34, S93, ADPA, Year 1)

*Their (non-TS) time is more flexible, at least they have more chances to go abroad and to join activities. We cannot join even if we wish to do so because our workloads are too heavy.*

(#38, S103, BM, Year 1)

As shown, the TS in this study often compared themselves with the non-TS. Unlike the non-TS who had lighter workloads overall and more time to engage in a wider variety of campus activities, the TS felt that they were restricted from participating in any extra-curricular activities owing to their heavy workloads and inflexible schedules.

#### 3.1.2. Change of Learning Approaches

Need to develop active and independent learning. Many TS deemed that the course content in university was more advanced, and the assessments were more difficult than their CC studies. In order to catch up, despite the difficulties, they needed to develop active and independent learning strategies and skills, such as self-directed learning.

*The course content is not as straightforward as before….When we were studying in CC, we had a better [academic] performance after we studied harder for the examination. …Now [in university] it is not enough just to study hard … I need to learn by myself [without help from teachers]. When I can’t understand the content delivered in lectures, I need to depend on myself or discuss with classmates.*

(#37, S100, BM, Year 1)

*We need to be self-directed in learning. It is not enough to spend more time reading. It is necessary to search for information and ask questions.*

(#1, S1, MDHS, Year 2)

Need to develop deep learning approach. The TS emphasized the need to demonstrate deep learning through understanding and applying the knowledge and practice.

*We have to do self-learning in a lot of courses. Skills are taught in laboratory sessions. We have to spend much more time to practise before we can master the skills. … Some knowledge can’t be internalized without understanding.*

(#1, S4, MDHS, Year 2)

*I had to demonstrate critical thinking when studying some courses. …I couldn’t handle assessments using rote learning only.*

(#35, S94, BM, Year 1)

*We are not deadline fighters. We will find ways to understand the subject content after each class to avoid accumulating the workload till the end of the semester.*

(#27, S74, BM, Year 1)

The process of transitioning from a passive to an active learner showed that the TS had the need, the ability and, to some extent, the flexibility to adapt to alternative learning approaches.

### 3.2. Challenges in Self-Identity and Social Experience

Studying in university is the goal of all the graduates from CC. The social environment plays a significant role in adjustment of TSs but at the same time it also brings challenges which they might not anticipate.

#### 3.2.1. Identifying Self with Reduced Sense of Competence

Sense of incompetence when compared with non-TS. The TS in this study achieved outstanding academic performances in their in CCs that allowed them to transfer to the university. However, it was common for them to express a sense of incompetence when comparing themselves with non-TS especially in terms of academic performance.

*We need to have a good foundation in order to have improvement. During our first year of study in the degree programme, we attended classes together with non-TS for the upper division courses. … so some weaker TS might have found it hard to catch up.*

(#32, S87, ATP, Year 2)

*Since we are TS, we might not be as clever as the non-TS. I am worried about the differences in our performance in assignments between us and the non-TS even though I put a lot of effort into my work. I feel inferior….*

(#10, S31, SV, Year 1)

Lack of confidence and sense of inferiority. The TS appreciated the superior performance of non-TS in their in-class performances. They considered their own performances to be poorer and realized that this might be related to their lack of confidence and sense of inferiority.

*I think we feel inferior so we believe they [non-TS] are better. I think we lack confidence … It is the reason for many issues …*

(#19, S59, MDHS, Year 3)

*I couldn’t get a high GPA because of my low intelligence. I feel helpless.*

(#30, S84, ATP, Year 1)

*They [non-TS] are much more confident than [us]... Even if they make mistakes, others might not notice them. I can see the difference.*

(#19, S59, MDHS, Year 3)

Uncomfortable when recognized as TS. Due to their sense of inferiority, the TS felt uncomfortable when recognized as TS in class. Some of them preferred to be integrated with non-TS in the learning environment. Despite their efforts, some believed they would not be able to integrate with non-TS.

*Some lecturers check the attendance of TS [only] during lectures. … They shouldn’t label us. We are not lazy nor ignoring our study.*

(#7, S26, MDHS, Year 2)

*We don’t want to be labeled that we failed in the public examination [for university entrance] and had to study in CC. We made a lot of effort, which most people don’t realize.*

(#7, S23, MDHS, Year 2)

#### 3.2.2. Restricted Social Circle

Staying within the circle of fellow TS. The majority of the TS expressed their tendency to stay within social circles with other TS as they had similar past experiences. They had limited interactions and reported having distant relationships with the non-TS.

*TS share similar experiences of failure in public examinations and thus the need to do an associate degree programme [in the CC], and then fighting hard to get into university. These similar experiences make it easier for us to get along with each other.*

(#6, S22, CSIT, Year 1)

*Non-TS have been studying [in the university] for two or three years; they have groups of friends already. It is normal that they enjoy staying with their friends. …We stick with our own transfer student groups as well, which is just normal.*

(#10, S30, SV, Year 1)

Having different goals and experience in their study when compared with the non-TS. The TS were accustomed to their degree aspirations shaping their mindsets and attitudes during their CC studies, and this was sustained in their degree studies. They believed that TS had different goals and experience in their university studies and were therefore unlikely to befriend each other:

*Non-TS and we have different goals. Unlike us, they do not focus too much on academic outcomes. It is difficult for us to befriend them since our goals are different.*

(#35, S94, BM, Year 1)

*Transfer and non-TS have different university experiences. Many of them [non-TS] join overseas exchange programs, but only a few TS can have this opportunity. They can do the placement for the work-integrated education course a number of times, but we can only do it once. They can join various [extra-curricular] activities, but we can only focus on our study.*

(#45, S121, SS, Year 2)

Limited chances to interact with non-TS. Although some TS reported being in the same classes as the non-TS, others had limited chances to meet their non-transfer classmates. This might also have contributed to their restricted social circles.

*In our first year in university, we were required to take courses different from those taken by the non-TS. …Then we only had the chance to study with non-TS in our second [i.e., final] year.*

(#46, S123, SS, Year 2)

*We are getting along well with other TS, but I don’t know any non-TS. Sometimes I meet some non-TS in one or two lectures, thus we do not have many chances to interact with one another.*

(#14, S45, MDHS, Year 1)

Comparison of TSs with their peers especially the non-TSs resulted in their sense of inferiority. Restricting their social circles and staying with fellow TSs enhanced their sense of comfort and positive feelings but may also hinder engagement in university life.

## 4. Discussion and Implications

In this study, transferring from two-year CCs to four-year universities brought about both academic and social challenges to CC TS. This corroborates previous quantitative studies of TS’ experiences in both Western [11,17] and Asian educational contexts [4,5,6,7].

### 4.1. Heavy Study Load Due to Limited Timeframe

The TS in this study perceived themselves as having heavy study loads, and this concern intensified when the timeframe for graduation was restricted. In order to graduate “on time”, they need to finish courses pre-assigned by their departments and sacrifice their interests in favor of courses that fit into their schedules; they might even need to give up campus activities and overseas exchange programs [9]. In other words, for the purpose of timely degree attainment, TS need to make choices about their course enrollments with a sense of urgency. This implies that the students might not always have the opportunity to realize their learning interests, which might be further detrimental to their learning experience on top of the heavy workload. This was, however, not a cause for complaint by the students in this study, perhaps owing to the emphasis on academic qualifications and post-secondary employability in Asian societies [37] that would reinforce their focus on their academic outcomes. Furthermore, while CC in the West aim at improving the affordability of tertiary-level schooling [41], those in Hong Kong are self-financed and the high tuition fees (than undergraduate tuition) can put considerable strain on the resources of students and/or their families [42]. The sooner they graduate and find jobs, the sooner they will benefit from this investment in academic qualifications [53]. This pragmatic and instrumental perception of university education, which has been prevalent in Hong Kong for two decades [54], justifies TS’ adaptive strategies and compliance to the post-secondary system and curriculum.

Despite that TS in the U.S. also view the completion of a degree programs as “opportunities for promotion and increased income, changing life situations” [55], they are not required to attain their degrees in two or three years but can instead take several years [56]. TS in Western educational contexts are sometimes referred to as “non-traditional students” who are more mature and enrolled part-time [30,57]. Meanwhile, even though those in Hong Kong study full-time and are required to graduate in a timeframe similar to that of a third-year non-transfer student, their heavier workloads and, as discussed later, their poor self-identities imply that this “alternative route to higher education” (p. 147) [46] merits rethinking and modification with regard to its positioning in the higher education system in Hong Kong in the long run. Meanwhile, in the short run, administrators and curriculum coordinators from CC and universities should work together towards solutions (e.g., setting up articulation agreements for improving credit transfer) that help reduce TS’ study loads and thereby alleviate their study-induced stress, and thus enhance their psychological well-being.

### 4.2. Flexibility in Learning Approaches

The academic achievement in CC supported these TS to be admitted to universities [46], which can be viewed as a TS capital [16]. However, due to their strong emphasis on academic performance “inherited” from their CC studies, these TS were aware of the need to develop alternative learning approaches. Our study showed that students had the need and ability to evolve from passive to active and independent learners. This indicates that these TS were not restrained by their previous training in CC, but could instead capitalize on their prior experience and demonstrated flexibility in adapting to the more dynamic university learning environment [58]. Aside from learning approaches, the TS in this study also exhibited appropriate learning attitudes, as evidenced by the perception that being “deadline fighters” is an improper practice. In order not to accumulate the workload until the end of an already overloaded semester, they preferred an understanding of the knowledge delivered after each class rather than rote-memorizing all the content at once. This “learning by understanding” tendency can be regarded as a form of deep learning [59]. The ability to adjust their conceptions of how learning should take place and their corresponding actions can counterargue negative opinions about CC TS being academically underprepared [22].

### 4.3. Poor Self-Identity throughout Transfer Experience

The TS’ poor self-identity was possibly linked, first, to their undesirable experiences of not fulfilling the requirements of the university entrance examination, and second, to having to enroll in CC to grab the “second chance” of getting into university [40]. Wong [40,60] found that, prior to transferring to university, CC students in Hong Kong already perceived themselves as “losers”, and that they were likely to carry this label with them even when they eventually gained admission to university. This self-perception of failure might have been internalized and turned into the sense of inferiority and incompetence, especially when compared to non-TS. Nonetheless, this study has shown that these TS still managed to handle the multiple sources of workload and even demonstrated the abilities to develop active and independent learning while aiming for timely degree attainment. Regardless of their academic achievement, they seemed to have magnified their negative pre-transfer experiences and ignored that they were successful CC graduates who were retained in the highly competitive environment, still persisting with their baccalaureate studies [61]. TS’ self-stigmatization, as indicated by our findings, show that intervention is needed to rectify their self-perceptions. This implies that various stakeholders in the university, including student affairs officers, teachers, and advisors, should constantly remind TS of their excellence, on such occasions as admission, staff–student consultative meetings, and informal gatherings.

### 4.4. Lack of Social Involvement

TS are often not as active in their social involvement as their non-transfer counterparts [13,62]. In Western educational contexts, their lack of social involvement is usually attributed to their personal backgrounds including being from low-income families [63] or having family and work responsibilities [20]. In Asian educational contexts, especially those dominated by Chinese societal values (e.g., Hong Kong), high school leavers are expected to start their baccalaureate education immediately before becoming part of the workforce [64]. Upon completing two years of CC education, the average age of TS in Hong Kong is therefore comparable to that of third-year non-TS; this similar age range should have allowed integration between the two groups of students as intellectual peers [65]. Nonetheless, the TS in this study were still inclined to interact only with other TS. On one hand, these qualitative findings further explain our quantitative results [4,5,6,7]—their self-stigmatizing sense of incompetence and inferiority already prevented them from reaching out to other students whom they deemed “stronger” but were in fact not necessarily so. This implies that more educational and psychological supports are needed to inform TS about the empirical evidence on how the academic performances of the two student subpopulations have been shown to be statistically similar [66,67]. On the other hand, as revealed by the perception of “goals and experience”, they might have stereotyped non-TS as caring less about their academic pursuits but more about their extra-curriculars. This could be a misunderstanding that has not been communicated between the two student groups, as some undergraduate students might still prioritize academic performance over other commitments [43]. With the absence of interactions between transfer and non-TS, the intervention of external parties such as administrators and teachers will play a vital role in helping to blend the two groups together. First, on the level of course arrangements, departments can arrange classes containing diverse student demographics, shown to facilitate teaching and learning in higher education [68]. Second, given that peer support is as important as teacher support [12], academic advisors should prompt non-TS to take the initiative in socializing with and assisting TS in both their academic and social integration. Last but not least, lecturers and tutors should avoid verbally expressing any labelling of TS, especially during classes and meetings [21].

### 4.5. Limitations

The current study adopted purposive sampling and a qualitative research design with a large sample size of 124 from nine disciplines. However, our sample was from a single institution, which hosts the most of TS in Hong Kong. To yield a more comprehensive picture of TS’ experience in Hong Kong, ongoing work is being conducted, analyzing interview data from other local institutions. Group interviews may hinder interviewees to reveal specific personal issues. In the study, seven individual interviews were conducted because of the availability of the interviewees. They allowed a more in-depth exploration of individual experience, but sharing among interviewees was not possible. This study aimed to synthesize the experience and challenges of TS in Hong Kong as one of the Asian educational contexts. The socio-cultural influence on their coping with the challenges and the required support deserve further exploration.

## 5. Conclusions

In this study, we explored the experiences of TS in an Asian educational context, which have received little attention in previous studies. The alternative pathway of TS entailed a different experience in their university studies when compared with non-TS. In the academic aspect, they faced a heavy workload from having to complete their studies within a limited timeframe for degree attainment with study-induced stress and had to develop learning approaches different from those used in CCs. In terms of their self-identity, they compared themselves with non-TS and felt less competent and inferior. Regarding their social experiences, they tended to bond with other TS and had limited chances of interacting with non-TS. As discussed, future policies and practices are warranted, not only to improve TS’ academic and social integration, but also to improve their self-perceptions and psychological well-being.

## Figures and Tables

**Table 1:** Interview guide.

**General Broad Opening Question**	
1.	Can you tell me about your experience studying in the university till now?
**Probing questions**	
1.	What do you think about your transition experience at the university? What were your major challenges?
2.	What were you particularly hoping to gain from the university study? What have you actually achieved?
3.	What learning method(s)/approach(es)/practice(s) do you use? Do you experience any challenges in learning?
4.	How have you found your relationships with other TS / non-TS? Do you experience any challenges?

**Table 2:** Categories and codes of the interview data.

Categories	Codes
Challenges in learning experience	
Heavy workload	Perception of foreshortened period of bachelor study
	Limited by insufficient credits transferred & extra workload from non-major courses
	Inflexible arrangement of time because of timetabling
	Dearth of time for extra-curricular activities
Change of learning approaches	Need to develop active and independent learning
	Need to develop deep learning approach
Challenges in self-identity and social experience	
Identifying self with reduced sense of competence	Sense of incompetence when compared with non-TS
	Lack of confidence and sense of inferiority
	Uncomfortable when recognized as TS
Restricted social circle	Staying within the circle of fellow TS
	Having different goals and experience when compared with the non-TS
	Limited chances to interact with the non-TS

## Data Availability

The data presented in this study are available on request from the corresponding author. The data are not publicly available due to privacy and ethical.
